# A case of multiple endo-epicardial connections between the left atrium and pulmonary vein treated by ultra-high-resolution mapping-guided laser balloon ablation

**DOI:** 10.1093/ehjcr/ytae277

**Published:** 2024-07-18

**Authors:** Yoshito Yamazaki, Seigo Yamashita, Michihiro Yoshimura, Teiichi Yamane

**Affiliations:** Division of Cardiology, Department of Internal Medicine, The Jikei University School of Medicine, Tokyo, Japan; Division of Cardiology, Department of Internal Medicine, The Jikei University School of Medicine, Tokyo, Japan; Division of Cardiology, Department of Internal Medicine, The Jikei University School of Medicine, Tokyo, Japan; Division of Cardiology, Department of Internal Medicine, The Jikei University School of Medicine, Tokyo, Japan

A 46-year-old man with paroxysmal AF underwent pulmonary vein isolation (PVI) using a third-generation LB system (LB-X3). After completion of continuous laser application using rapid mode (13 W) at the left superior PV (LSPV) ostium, residual PV potentials were detected that could not be eliminated by additional laser application (15 W). The 3D-EAM system (Rhythmia™; Boston Scientific, MA, USA) showed double potentials along the prior laser line, whereas focal activation was found away from the laser line (*Figure 1A*). These findings indicated the presence of an endo-epicardial connection (EC) between the left atrium (LA) and LSPV. The PV potential sequence changed after touch-up laser application (10 W) at the EC site under the guidance of the inner view (*Figure 1A*), and remap revealed another EC at the bottom anterior of the LSPV. Residual LSPV potentials disappeared in real time immediately after Rhythmia™-guided touch-up laser application (10 W) at the second EC site (*Figure 1B*). Although not proved, it seems reasonable to consider the presence of two distinct ECs between the LA and LSPV from the fact that the second EC possibly via the Marshall bundle has appeared after the elimination of the first EC. To our knowledge, this is the first report of multiple ECs after LB-guided PVI that were successfully isolated by spot laser applications in real time under the Rhythmia™ system inner view.

**Figure 1:**
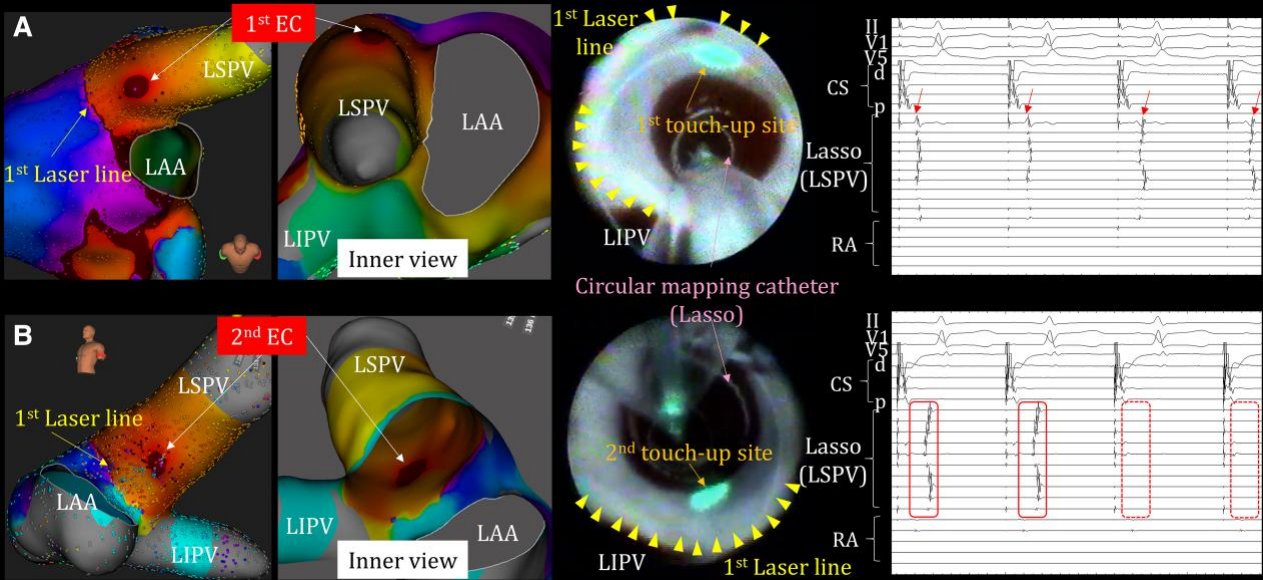
(*A*) During distal coronary sinus (CS) pacing, the activation sequence of pulmonary vein potentials (PVPs) on a circular mapping catheter (Lasso, Biosense Webster, CA, USA) located distal to LSPV has changed (arrow in the right electrogram) just after Rhythmia™ guided spot laser application at the first EC site. (*B*) PVPs were eliminated (dotted box line) by Rhythmia™ guided spot laser application at the second EC site. Of note, spot laser application sites were distally away from the first laser line (yellow triangles) in the endoscopic view, where were identical to EC sites in the Rhythmia™ inner view. CSd, distal coronary sinus; CSp, proximal coronary sinus; EC, endo-epicardial connection; LAA, left atrial appendage; LIPV, left inferior pulmonary vein; LSPV, left superior pulmonary vein; RA, right atrium.

##  

**Consent:** Informed consent was obtained from the patient for publication.

**Funding:** None declared.

## Data Availability

The data underlying this article are available in the Dryad Digital Repository.

